# Experimental Study on Hydrogen-Induced Crack Propagation of X80 Steel Under Alternating Pressure Fluctuations

**DOI:** 10.3390/ma18050947

**Published:** 2025-02-21

**Authors:** Ailing Wang, Baogang Wang, Ruijing Jiang, Ruyu Nie, Gan Cui, Jianguo Liu, Yi Zhang, Hao Zhang, Xiao Xing

**Affiliations:** 1 College of Pipeline and Civil Engineering, China University of Petroleum (East China), Qingdao 266580, China; 2 Southwest Pipeline Company, Pipe-China, Chengdu 637400, China; 3 CNPC Research Institute of Safety and Environment Protection Technology, Building 1, Beijing 102206, China; 4 Shengli Offshore Petroleum Engineering Technology Inspection Co., Ltd., Sinopec, Dongying 257000, China; 5 Department of Chemical and Materials Engineering, University of Alberta, Edmonton, AB T6G 1H9, Canada

**Keywords:** X80 steel, hydrogen-blending transportation, hydrogen embrittlement, fatigue crack propagation

## Abstract

When hydrogen is transported in a pipeline, the fatigue loading in the pipeline will enhance hydrogen accumulation and diffusion, thus increasing the risk of hydrogen-induced fracture. In this study, specimens are subjected to cyclic loading within an autoclave, where hydrogen gas pressure is varied to examine its impact on fatigue crack growth. The influence of hydrogen pressure and stress variations on the fatigue crack growth rate is investigated. The findings show that as hydrogen pressure increases, the crack growth rate also rises, and at 3 MPa hydrogen pressure the rate is elevated by one order of magnitude compared to that in air, reaching 10^−2^ mm/cycle. In hydrogen, the fatigue crack propagation rate decreases with increasing loading frequency. When the frequency is 0.02 Hz, the crack propagation rate reaches a maximum of 10^−2^ mm/cycle, whereas at 0.5 Hz, the fatigue crack propagation rate is generally below 10^−3^ mm/cycle. With the maximum stress held constant during cyclic loading, the fatigue crack growth rate increases as the stress range widens, and when the stress ratio reaches 0.5, the crack propagation rate can increase to a maximum of 10^−1^ mm/cycle. Based on these experimental results, a predictive model is proposed to estimate the crack growth rate under different hydrogen pressures and loading conditions, and the average relative errors of predictive values and experimental data are limited below 10%.

## 1. Introduction

The consumption of fossil fuels, including coal, oil, and natural gas, has contributed to rising carbon dioxide emissions and intensified the greenhouse effect. Currently, researchers are focusing on advancing and utilizing renewable energy sources, such as wind and solar power [1]. However, the energy from wind and solar is difficult to store and transport. To achieve large-scale green energy storage and transportation, hydrogen generated from an electrolysis reaction is determined to be the most promising energy carrier [2,3,4].

Hydrogen transportation is a primary section of the hydrogen energy utilization process. Typically, hydrogen can be transported using methods such as tube trailers, liquid hydrogen tankers or ships, and hydrogen pipeline systems. Among these, pipeline transportation of high-pressure hydrogen offers superior economic efficiency and is more suitable for large-scale, long-distance hydrogen delivery [5]. However, building new hydrogen pipeline networks will bring high costs and geographical limitations. Therefore, repurposing existing natural gas pipelines for the blended transportation of natural gas and hydrogen has emerged as a viable solution [6,7]. Hydrogen gas could disassociate into atomic hydrogen and then enter the pipeline. The hydrogen atoms could diffuse in the tetrahedral sites in the body center cubic (BCC) structure of the pipeline and cause hydrogen embrittlement (HE) [8,9]. Moreover, X80 steel, which is the commonly used metal in gas pipelines, is highly susceptible to hydrogen embrittlement. HE can lead to pipeline rupture and system equipment failure during operation [10,11]. Due to the start-up and shutdown of the compressor, the volume adjustment caused by market demand, and the change in the external environment, the pipeline is subjected to the alternating load or cyclic load. The interaction between high-pressure hydrogen and the alternating load in the hydrogen-blending transportation pipeline will accelerate the fatigue crack growth rate of pipeline steel [12,13].

Currently, numerous studies have investigated the impact of hydrogen concentration on the fatigue crack propagation behavior of pipeline steel. One widely employed method for generating hydrogen in experiments is electrochemical hydrogen charging, where hydrogen atoms are produced through cathodic reactions [14,15]. In contrast, the operating conditions in hydrogen-blending pipelines involve a high-pressure hydrogen environment, where hydrogen atoms primarily result from the process of hydrogen molecules adhering to and breaking apart on the pipeline’s steel surface [16]. The atomic hydrogen concentrations of the two hydrogen-generating methods are very distinct [17]. Also, the alternative loadings in the pipeline complicate the hydrogen diffusion and accumulation process [18]. Hence, most of the previous research results cannot be directly applied to hydrogen-blending pipelines. The impact of hydrogen pressure on crack propagation in pipeline steel remains unclear, and there is no precise model to quantify its effect on the microscopic crack growth. L. Briottet et al. [19] suggested that, at low hydrogen pressures, hydrogen does not accelerate the propagation of fatigue cracks. When the hydrogen pressure is below 0.5 MPa, the crack growth of the specimen is similar to that observed in the air. Fernando et al. [20] found that the fatigue crack initiation rate in the air was higher than that under low hydrogen pressure conditions where the hydrogen pressure is below 3.45 MPa. Bryan et al. [21] conducted fatigue crack growth experiments under hydrogen pressures of 0.7 MPa, 20.7 MPa, and in the air. They found that, at a hydrogen pressure of 0.7 MPa, the fatigue crack growth rate in hydrogen was slower compared to that in the air. Xing et al. [22] demonstrated that the cyclic load in the pipeline continued to accumulate hydrogen atoms towards the crack tip. Although the concentration of hydrogen atoms in the pipeline is low under moderate hydrogen pressures, these atoms can still significantly influence the fatigue crack growth rate through continuous accumulation. Slifka et al. [23] observed that the fatigue crack growth rate of pipeline steel was notably higher in hydrogen compared to in the air at hydrogen pressures of 1.7 MPa, 7 MPa, and 21 MPa. Zhou et al. [24] studied how hydrogen influences the tensile characteristics of X80 pipeline steel in a nitrogen–hydrogen mixture, with hydrogen volume fractions of 0%, 1%, 2.2%, and 5% under a total pressure of 12 MPa. Their results revealed that hydrogen had minimal influence on the tensile and yield strengths of the specimens. Based on the above discussion, a contradiction exists on whether comparatively low-pressure (5 MPa or below) hydrogen gas could significantly degrade the physical properties of the metals.

Pipeline steel is always under the influence of alternating loads. The factors of the alternating load mainly include the frequency *f* and stress ratio *R*. In the air, as the stress fluctuation range remains unchanged, the frequency does not affect crack propagation [25]. Suresh and Ritchie [26] investigated the impact of hydrogen on fatigue crack growth and found that crack propagation in specimens was affected by the loading ratio, loading frequency, and hydrogen pressure. However, they did not establish a predictive model for crack propagation. Somerday et al. [27] investigated how cyclic loading frequency and load ratio influence hydrogen-induced fatigue crack growth. Their results showed that a lower cyclic loading frequency or a higher *R* ratio postponed the initiation of hydrogen-accelerated crack growth to greater Δ*K* levels. However, the reliability of the predictive model remains unconfirmed. W. Chen et al. [28,29,30] observed that within a frequency range of 5.0 × 10^−1^ Hz to 10^−5^ Hz, the crack growth rate remained unchanged in the air but increased with decreasing loading frequency in a hydrogen environment. Based on experimental data, they developed a crack propagation prediction model. However, their study was conducted under electrochemical hydrogen charging conditions and did not account for the effects of stress ratio or hydrogen pressure. Shinko et al. [31] analyzed the influence of pressure and loading frequency on crack propagation in a gaseous hydrogen environment. However, their predictive model lacked a detailed characterization of the corresponding force ratio. Holbrook et al. [32] demonstrated that in hydrogen the crack growth rate exhibited a consistent rise with an increasing *R* ratio. However, their model lacked the ability to quantify the influence of loading frequency. Yamabe et al. [33] performed experiments on low-carbon steel specimens under varying pressures and loading frequencies. To quantify the acceleration of fatigue crack growth, they analyzed hydrogen trapping behavior and diffusivity in pre-strained samples. However, their quantitative model did not incorporate the influence of stress ratios. These studies illustrate the impact of varying loading conditions on the crack growth rate, particularly under electrochemical hydrogen charging conditions.

Even though several prediction models on the crack propagation rate of hydrogen-induced cracking have been developed, no predictive model is perfectly applicable for the fatigue crack growth rate of hydrogen transportation pipelines. Therefore, it is necessary to design cyclic loads according to the actual pressure fluctuations of pipelines and establish a fatigue crack growth rate model of pipeline steel under the combined effect of hydrogen concentration and cyclic loads. This is crucial for ensuring the safe operation and longevity assessment of hydrogen-blended pipelines.

## 2. Materials and Methods

### 2.1. Materials and Devices

**Figure 1:**
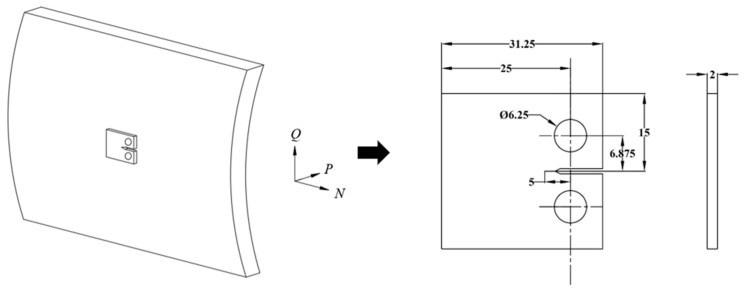
Dimensions of CT specimen, in mm.

**Figure 2:**
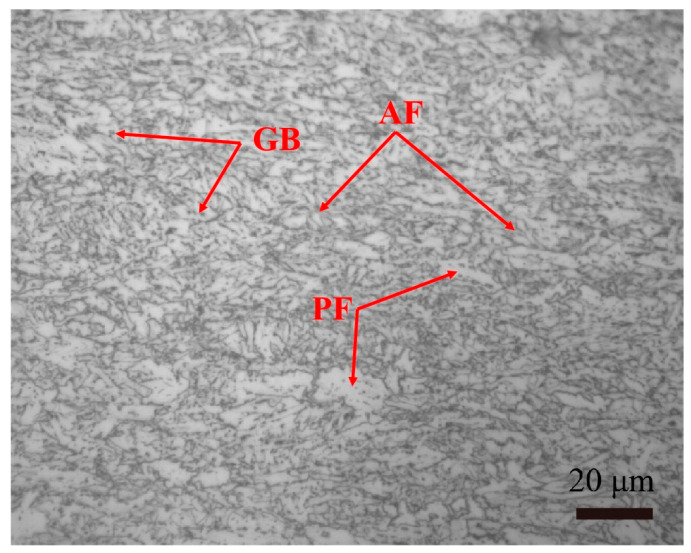
SEM micrograph of X80 Steel.

The specimen used in the tests is constructed from API Spec 5L X80 pipeline steel. To analyze the types and distribution of elements, scanning electron microscope (SEM) images were captured at magnifications of 1000 and 2000 using an Apeo S Field emission scanning electron microscope (Thermo Fisher Scientific, Waltham, MA, USA). Line and area scans were performed on the SEM images, and energy dispersive spectroscopy (EDS) was utilized to analyze the elemental composition based on the acquired spectra, as shown in Table 1. The tensile strength of the steel is 659 MPa, and the yield strength is 575 MPa. The specimens are standard compact tensile (CT) specimens, and the dimension of each specimen is shown in Figure 1. Specimen size selection and subsequent experimental methods take reference from standard ASTM E647 [34]. The thickness of the specimen is selected to be 2 mm, which is a minimum value in the standard. Specimens with a thickness of 2 mm comply with the standard ASMT E647; this selection can shorten the test schedule, but it may cause higher fracture toughness. Before the experiment, the steel is cut into 5 mm × 5 mm × 2 mm specimens. Then, it is ground by sandpaper with 600, 800, and 1200 grits, and polished using a polishing cloth with diamond polishing paste. Afterwards, the specimen is placed in a nitric acid alcohol solution with a volume fraction of 4%. The Zeiss Axio Imager A2m metallographic microscope is used for observation. The microstructure is mainly composed of quasi-polygonal ferrite (PF), acicular ferrite (AF), and granular bainite (GB), as Figure 2 shows.

**Figure 3:**
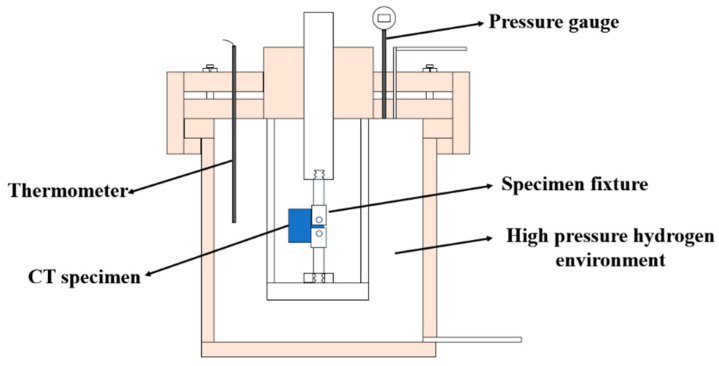
Schematic illustration of high-pressure hydrogen environmental fatigue experimental device.

A WDML-50 microcomputer-controlled slow-strain–stress corrosion testing machine equipped with a high-pressure hydrogen reactor is used in this study. The vessel of the high-pressure gas reactor is made of Hastelloy C-276, which has excellent resistance to hydrogen embrittlement and corrosion, as shown in Figure 3. A gas inlet and outlet adjustment system is used to control the pressure in the autoclave. The purity of the hydrogen used in the experiment was 99.999%. Due to the high risk of hydrogen leakage, a hydrogen concentration detector, alarm, and an explosion-proof variable-frequency exhaust machine are installed. The reaction kettle is filled with hydrogen at a specific pressure during the experiment, and the reaction kettle applies fatigue load to the specimen along with the movement of the beam at the lower part of the testing machine.

### 2.2. Experimental Method

**Figure 4:**
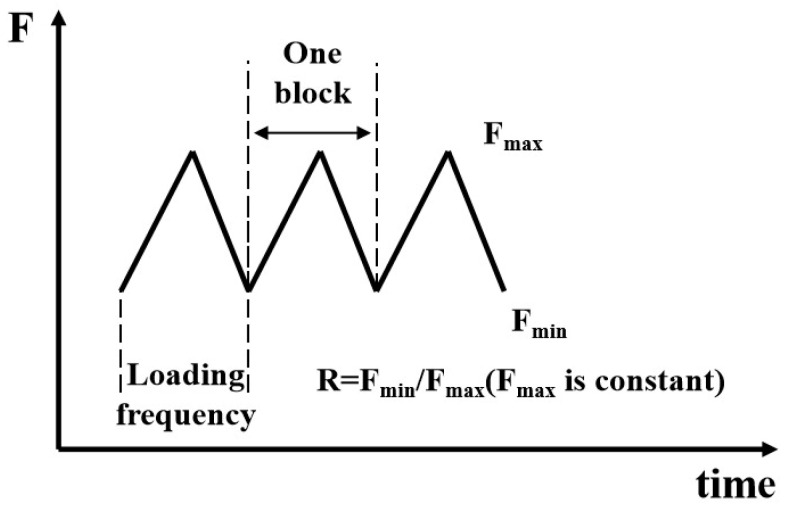
Fatigue load applied to the specimen.

Firstly, the specimen was ground with sandpaper and then polished using a polishing cloth with diamond polishing paste. The specimen was subsequently cleaned using a mixture of absolute ethanol and acetone in an ultrasonic cleaner to remove moisture and grease from its surface, followed by drying and sealing for storage. Before the fatigue test, the specimen was prefabricated with fatigue cracks on the QBG-100 high-frequency fatigue testing machine, in which a 2 mm crack was generated. The autoclave is filled with 1 MPa of nitrogen to remove the air and then the autoclave is purged three times with 1 MPa hydrogen gas to ensure the complete removal of nitrogen. After all the nitrogen was replaced, the gas outlet valve was shut down. The hydrogen gas was slowly introduced until the required pressure was reached. The temperature in the reactor was adjusted to 20 °C using a constant temperature circulation system. After the temperature and pressure were stabilized, the fatigue load was applied through the testing machine programming. Figure 4 shows the schematic of the fatigue loads. The standard for fatigue crack growth rate testing is referenced to ASTM E647.

The crack length was measured using the compliance method, and according to the standard “GB/T 6398-2017 Metallic materials—Fatigue testing—Fatigue crack propagation methods” [35], the crack length is calculated according to the following equation:

(1) $$U_{x}={\left\{{\left[\frac{BEV_{x}}{F}\right]}^{1/2}+1\right\}}^{-1}$$

where *U_x_* represents the dimensionless compliance, *B* is the specimen thickness, *E* denotes the modulus of elasticity, *V_x_* is the displacement observed at the designated measurement point, and *F* is the force loaded on the specimen.

The correlation between compliance and normalized crack length is expressed as follows:

(2) $$a/W=C_{0}+C_{1}U_{x}+C_{2}U_{x}^{2}+C_{3}U_{x}^{3}+C_{4}U_{x}^{4}+C_{5}U_{x}^{5}$$

where *a* is the crack length, *W* is the width of the specimen, and *C*_0_~*C*_5_ are constants, which are related to *V_x_*, namely, related to the measurement position of the specimen. The *C* values are shown in Table 2.

After obtaining the crack lengths corresponding to different cycles, the crack growth rate is calculated using the secant method. The formula is as follows:

(3) $$\frac{da{\left(j\right)}_{avg}}{dN}=\frac{\left[a_{j}-a_{\left(j-1\right)}\right]}{\left[N_{j}-N_{\left(j-1\right)}\right]}$$

where [*a_j_* − *a* _(*j*−1)_] is the crack increment.

The stress intensity factor in the *j*-th cycle is analyzed using the following equation [36]:

(4) $$\Delta K_{j}=\frac{\Delta F}{BW^{1/2}}g\left(\frac{a_{j}}{W}\right)$$

(5) $$g\left(\frac{a_{j}}{W}\right)=\frac{\left(2+\alpha \right)\left(0.886+4.64\alpha -13.32\alpha^{2}+14.72\alpha^{3}-5.6\alpha^{4}\right)}{{\left(1-\alpha \right)}^{3/2}}$$

where Δ*F* is the change in the force applied to the specimen, and *g* ($\frac{a}{W}$) is the specimen geometry factor in which α = *a*/*W* ranges from 0.2 to 1.

Through the above calculation, the crack growth rate and stress intensity factor can be obtained, and the fatigue crack growth curve can be depicted.

## 3. Results and Discussion

### 3.1. Effect of Hydrogen Pressure on Crack Growth Rate

**Figure 5:**
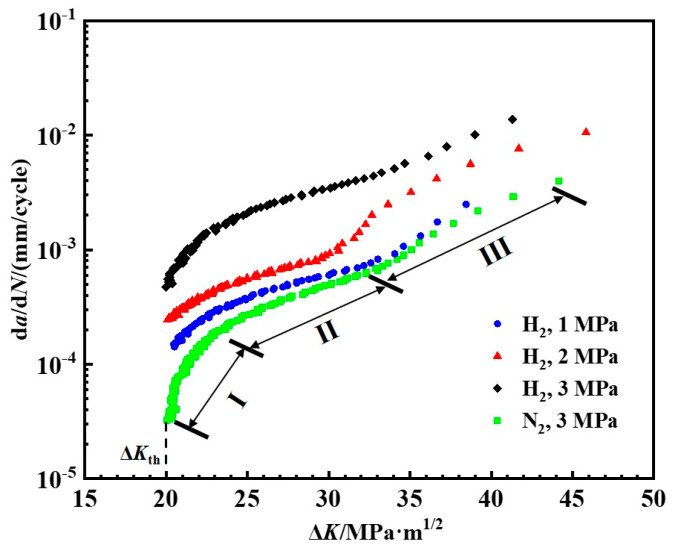
Fatigue crack growth curve of pipeline steel under different hydrogen pressures.

Fatigue crack propagation experiments were performed at varying hydrogen pressures to construct the crack growth curve. The maximum applied force *F_max_* for the fatigue load was 3.8 kN, with a stress ratio of 0.6 and a loading frequency of 0.1 Hz. The tests were performed at hydrogen pressures of 1 MPa, 2 MPa, and 3 MPa, in sequence. Figure 5 shows the experimental results and data. Evidently, the crack growth rate of X80 pipeline steel is substantially enhanced by hydrogen pressure. Moreover, the crack growth rate increases one order at a moderate hydrogen pressure of 3 MPa compared with that in the nitrogen environment. The current tests suggest that the HE effect is not negligible, even under a comparatively low hydrogen pressure or under a moderate pressure. Hydrogen atoms can easily diffuse through the steel lattice, especially at defects such as crack tips or grain boundaries. When hydrogen accumulates in these regions, it weakens the atomic bonding, leading to embrittlement of the material [37]. In a hydrogen environment, the crack propagation rate of the steel increases significantly. The steel used in this study is X80 steel. However, other types of pipeline steels, such as X65 and X52 steels, are also susceptible to hydrogen embrittlement under the influence of hydrogen gas [38,39].

Figure 5 demonstrates that the complete fatigue crack propagation curve approximates the “S” shape in the nitrogen. The “S” shape curve can be categorized into three distinct regions. The initial stage is the initiation phase of fatigue cracks, also known as the low-rate region. Even though the crack growth rate in this part is low, it increases sharply with the increment of the stress intensity amplitude Δ*K*. A critical threshold, Δ*K*_th_, exists below which the crack growth rate approaches zero. Under current experimental conditions, the Δ*K*_th_ in the nitrogen environment is 20 MPa·m^1/2^. However, there is no obvious fatigue growth threshold in the hydrogen environment, suggesting that hydrogen significantly lowers the fatigue crack growth threshold Δ*K*_th_. Thus, cracks enter the second stage at a lower stress intensity. The second stage is shown as a straight line and possesses middle-rate crack propagation where the Δ*K* value ranges from 22.2 to 33.1 MPa·m^1/2^. This section could be fitted by using the Paris law. Cracks undergo rapid propagation until they fracture in the third stage, in which the d*a*/d*N* becomes more significant than that in the second stage.

### 3.2. Effect of Frequency on Crack Growth Rate in Hydrogen Environment

**Figure 6:**
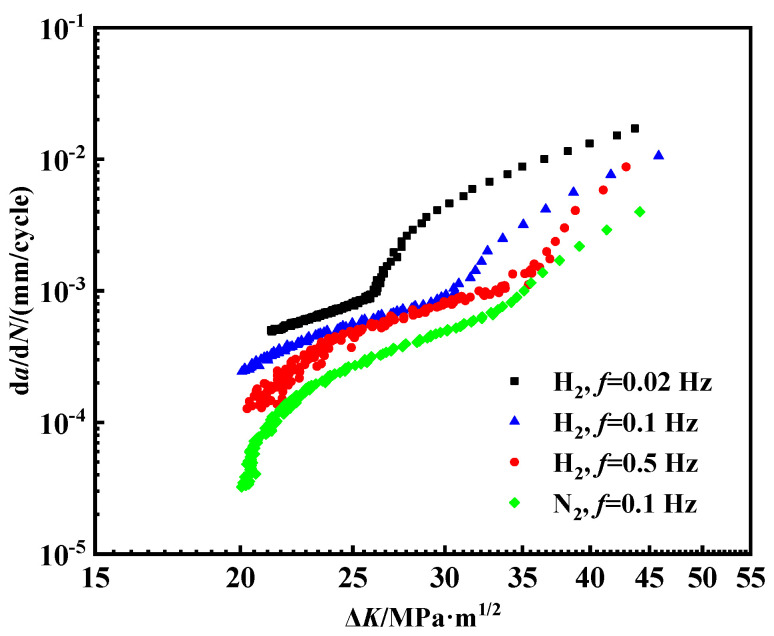
Fatigue crack growth curve of pipeline steel under different frequencies.

In the field operations, the frequency of the fatigue loads in pipelines typically ranges from 1 × 10^−5^ Hz to 0.1 Hz [40]. Therefore, the frequency range for the laboratory experiments was selected to be between 1.25 × 10^−3^ and 0.1 Hz, taking into account the time constraints. In air, the effect of loading frequency on the crack growth rate is negligible [25]. The impact of loading frequency on the fatigue crack growth rate was further investigated under a hydrogen pressure of 2 MPa. The maximum applied load, *F*_max_, was 3.8 kN, with a stress ratio *R* of 0.6. Figure 6 shows the corresponding results.

**Figure 7:**
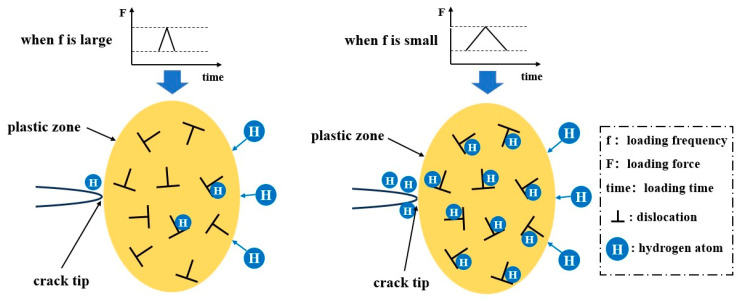
Schematic diagram of hydrogen diffusion in the plastic zone at a crack tip.

The experimental findings indicate that the frequency exerts a considerable influence on the crack growth rate in the hydrogen environment. Generally, as the frequency increases, the crack growth rate per cycle decreases. Additionally, the crack growth rates in the hydrogen environment at various frequencies are higher than those observed in the nitrogen environment. The frequency dependence observed is mainly due to the hydrogen equilibrium concentration within the plastic zone. As shown in Figure 7, hydrogen atoms accumulate in the plastic zone ahead of the crack tip, facilitating the diffusion of excess hydrogen to the crack tip and accelerating the crack propagation. At lower frequencies, the longer loading duration allows hydrogen atoms to diffuse more effectively towards the crack tip, resulting in a higher local concentration of hydrogen and thus promoting crack growth with each cycle. Conversely, at higher frequencies, hydrogen atoms lack sufficient time to be transported to the crack tip [41]. A larger plastic zone can alleviate stress concentration and trap hydrogen atoms, thereby slowing down the crack growth rate. Only when the hydrogen atom concentration within the plastic zone reaches saturation can the hydrogen atoms reach the free surface at the crack tip, promoting crack propagation.

### 3.3. Effect of Stress Ratio on Crack Growth Rate in Hydrogen Environment

**Figure 8:**
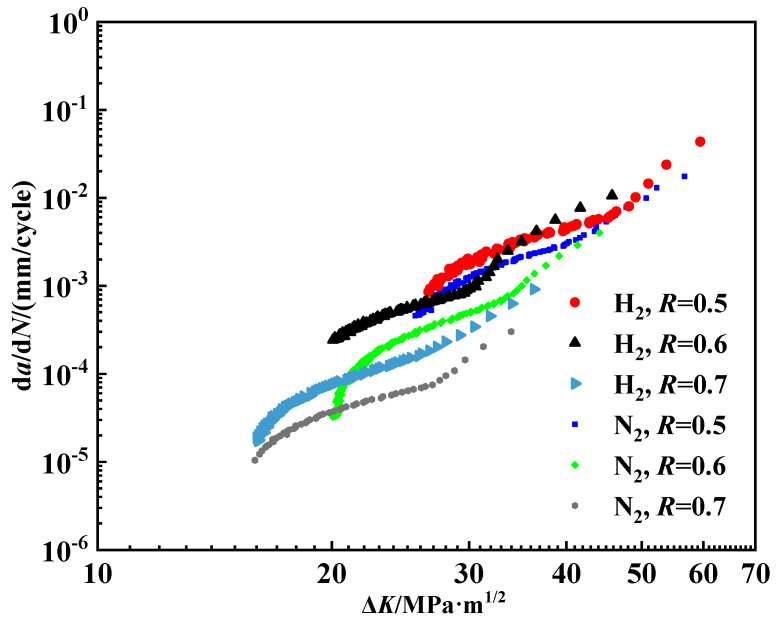
Fatigue crack growth curve of pipeline steel under different stress ratios.

The amplitude of the internal pressure fluctuation of the pipeline will change during operation, leading to a variation in the stress ratio *R*. To study the effect of the *R* value on the fatigue crack growth behavior of pipeline steel in a hydrogen environment, the fatigue crack growth rates are measured when *R* values are set as 0.5, 0.6, and 0.7 in a 2 MPa hydrogen environment and a nitrogen environment. The maximum force value *F_max_* is set as 3.8 kN and remains unchanged in the test. The change in the stress ratio is carried out by changing the minimum force value. The loading frequency is set to 0.1 Hz, and Figure 8 shows the results.

As Figure 8 depicts, while the initial crack length remains constant, variations in force range result in different starting points for the curve. Overall, the influence of the stress ratio on the fatigue crack growth behavior of X80 steel exhibits a similar trend in both nitrogen and hydrogen environments. Specifically, the crack growth rate consistently increases as the R value decreases. Moreover, the crack growth rate in a hydrogen environment is generally higher than that observed in nitrogen.

### 3.4. Fracture Analysis

**Figure 9:**
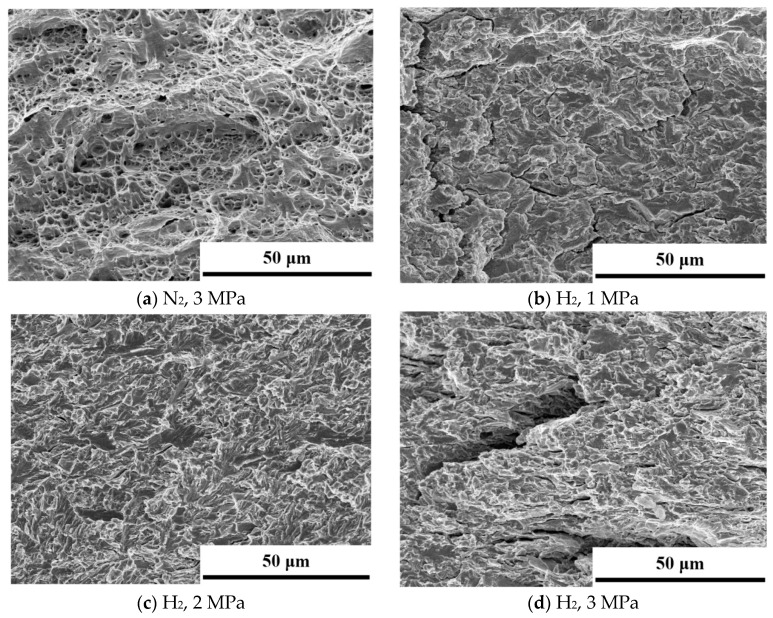
SEM micrographs of fracture as the Δ*K* equals 27 MPa·m^1/2^.

**Figure 10:**
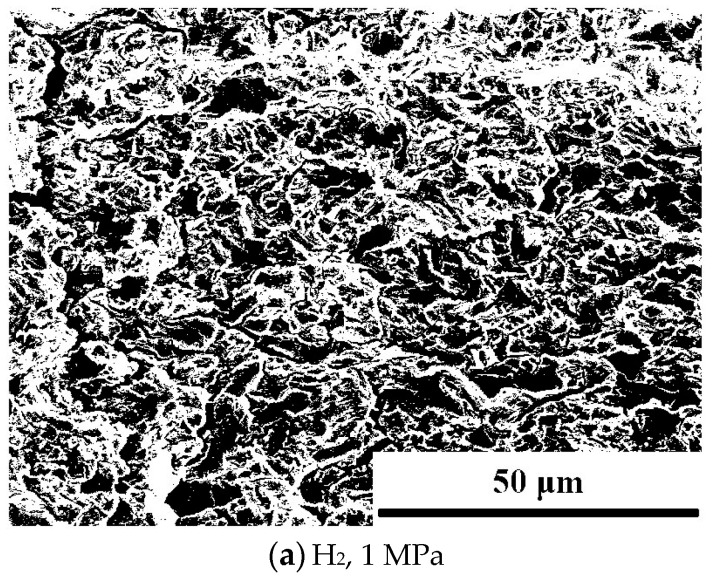

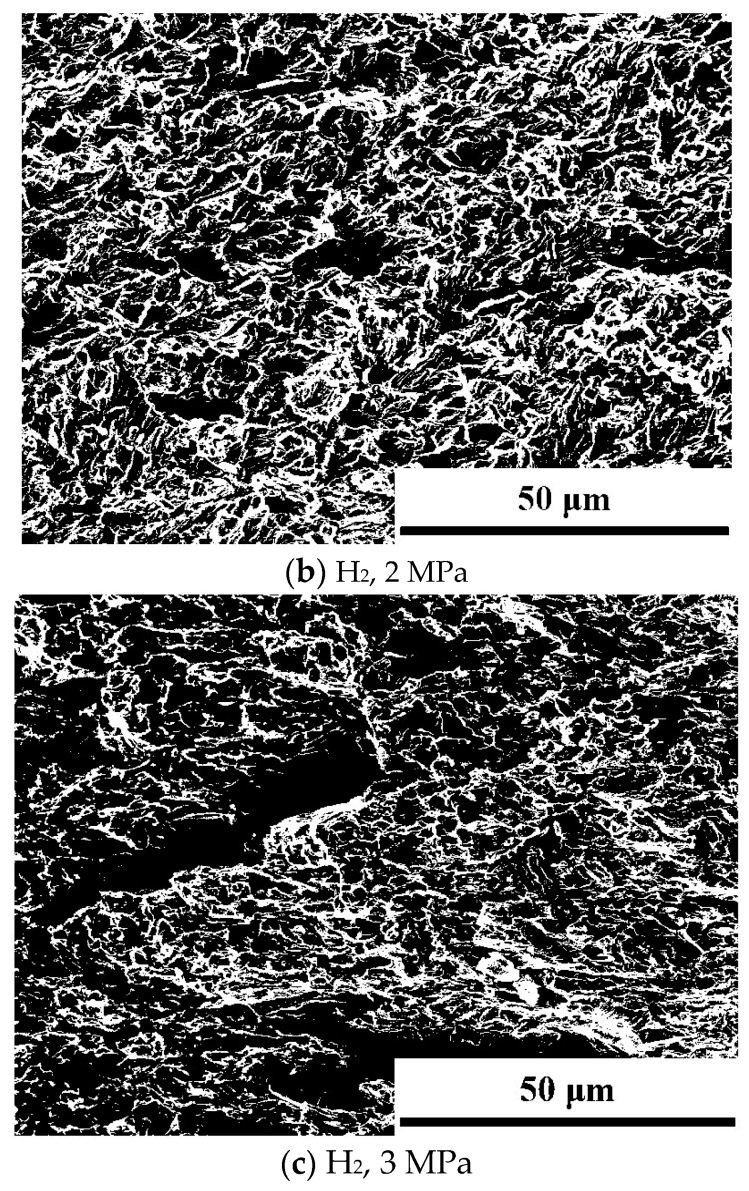
Processed SEM micrographs of fracture under different hydrogen pressures.

The fracture surfaces at different hydrogen pressures are observed by SEM, as Figure 9 shows. The maximum fatigue load force, *F_max_*, is 3.8 kN, the stress ratio *R* is 0.6, and the loading frequency *f* is 0.1 Hz. In nitrogen, the fracture surface is filled with dimples, showing a typical ductile fracture morphology. Under hydrogen pressures of 1 MPa and 2 MPa, the fracture morphology is similar, and the river pattern fracture feature is identified. Also, there are many tear edges. At a hydrogen pressure of 3 MPa, the obvious step-like secondary cracks appear, which is a typical brittle feature. Conclusively, there are no obvious dimples, and the fracture is full of tear edges, resulting in secondary cracks. SEM images under different hydrogen pressure conditions were processed using the ImageJ software (https://imagej.net/ij/), as Figure 10 shows. The brittle fracture areas, including secondary cracks and cleavage fracture platforms, were marked in black, while the other regions were marked in white. ImageJ was then used to calculate the proportion of the brittle fracture areas relative to the total area. At a hydrogen pressure of 1 MPa, this proportion was 47.56%, while at 2 MPa and 3 MPa, it increased to 59.39% and 69.08%, respectively, showing a clear upward trend. The results indicate that even at moderate hydrogen pressures, hydrogen has caused significant hydrogen embrittlement (HE) in the material under cyclic loading. Hydrogen-induced surface cracks do not necessarily lead to brittle fracture. Under certain stress conditions, surface cracks may cause subsequent fracture to be more of a tearing nature, exhibiting ductile fracture characteristics, with brittle features only observed at the initiation site [42]. In this study, the fracture surfaces of specimens exposed to hydrogen exhibited brittle fracture characteristics as the loading method used was fatigue loading. With each load cycle, hydrogen atoms continue to accumulate at the crack tip, embrittling the material at the crack front and ultimately leading to brittle fracture.

**Figure 11:**
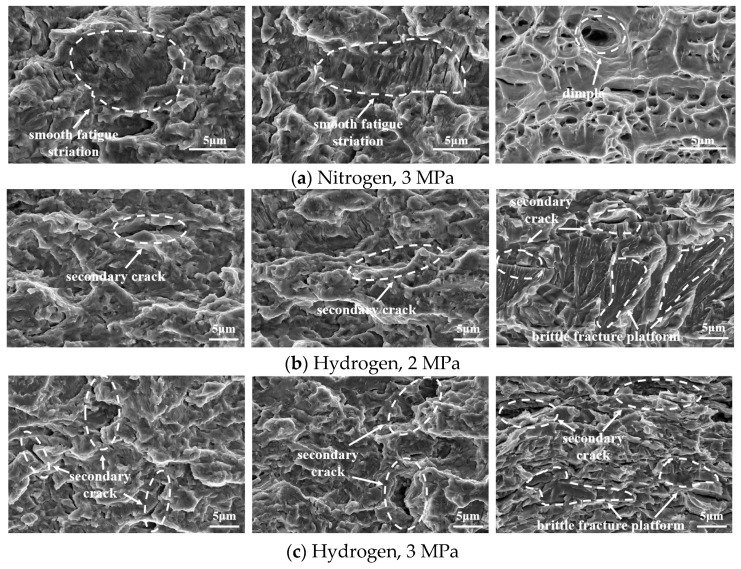
SEM micrographs of fracture along the crack propagation path. From (**right**) to (**left**), the images correspond to the initial, middle, and final stages of crack propagation.

Figure 11 shows the SEM micrographs of the fracture surfaces of the CT specimens along the crack propagation path. From left to right, the ∆*K* value approximates 22 MPa·m^1/2^, 31 MPa·m^1/2^, and 45 MPa·m^1/2^, corresponding to the early, intermediate, and advanced stages of fatigue crack growth, respectively. Under nitrogen conditions the fracture surfaces at each stage exhibit ductile characteristics, with clearly visible smooth fatigue striations in the initial and middle stages. It is evident that as the crack propagates, the spacing between fatigue striations gradually increases, correlating with an accelerated crack propagation rate. Conversely, in a hydrogen gas environment all fracture surfaces display brittle features, where the river pattern feature appears. As hydrogen exists, the increasingly prominent secondary cracks appear during the crack propagation. The third image from the left illustrates the phase of rapid crack propagation, wherein the specimen fails instantaneously as the remaining area along the crack propagation direction is unable to withstand the load. Under nitrogen conditions, this phase is characterized by ductile fracture with visible dimples. In the later stage of crack propagation, HE becomes more pronounced than in the stable phase. This is primarily due to the accumulation of tensile stress at the crack tip, which facilitates localized hydrogen concentration. Thus, sizeable brittle fracture platforms are observed in the final stages of the hydrogen environment.

## 4. Establishment of a Fatigue Crack Propagation Model

A quantitative model for the fatigue crack growth rate of pipeline steel in hydrogen has not yet been established. In the stable crack propagation phase, the crack growth rate (da/dN) exhibits a linear correlation with the applied stress intensity factor. The Paris law was established to predict crack growth based on an extensive array of experiments [43]. This equation effectively predicts fatigue crack growth rates within the stable propagation region. The formula is expressed as follows:

(6) $$\frac{da}{dN}=C{\left(\Delta K\right)}^{m}$$

where *C* and *m* are material-dependent constants derived from experimental data fitting. However, the Paris law has some limitations, as it does not consider the initial crack initiation phase or the rapid propagation phase. Furthermore, it does not account for the influences of maximum stress intensity, loading frequency, or hydrogen pressure on the crack growth rate.

**Figure 12:**
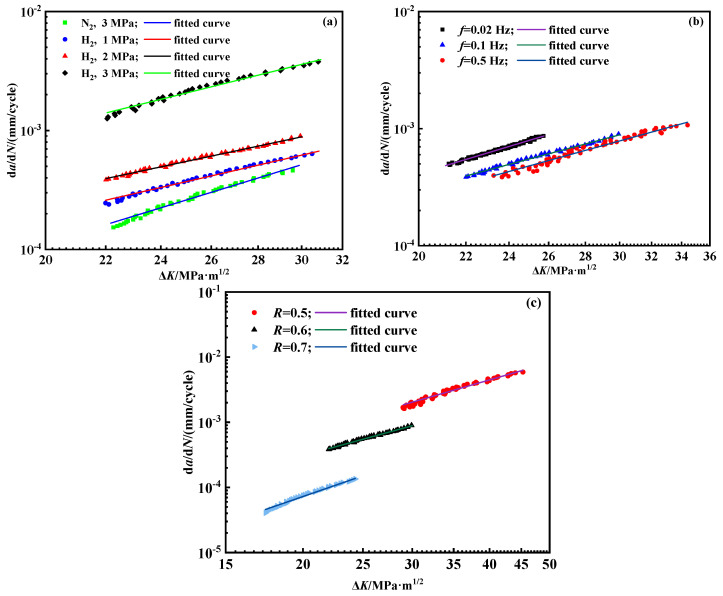
Fitting of d*a*/d*N*-Δ*K* curves under different conditions. (**a**) Curve fitting under different hydrogen pressures; (**b**) Curve fitting under different frequencies; (**c**) Curve fitting under different stress ratios.

Currently, the Paris equation is the most widely applicable fatigue crack prediction model. The curve during the stable crack propagation stage was fitted using the orthogonal distance regression method. At a hydrogen pressure of 2 MPa, the fitted curves at different frequencies and stress ratios are depicted in Figure 12. The values of *C* and *m* in the fitted Paris formula for pipeline steel under different conditions are presented in Table 3. All the fitted values in Figure 11 are predicted by Equation (6).

Table 3 reveals notable variations in the parameters *C* and *m* of the Paris formula across different operating conditions. Although the Paris formula has good accuracy and a simple form, it needs to be modified when the loading conditions change. The poor versatility limited the application of the Paris law.

The Walker [44] model is based on the Paris law and considers the impact of stress ratio *R*. It is expressed by the following equation:

(7) $$\frac{da}{dN}=C{\left[\frac{\Delta K}{{\left(1-R\right)}^{n}}\right]}^{m}$$

where *n* and *m* are constants and the correction of the stress ratio is involved here.

Cheng and Chen [41] developed a model correlating corrosion and crack propagation to describe the impact of hydrogen embrittlement on the fatigue crack growth of pipeline steels in a gaseous hydrogen environment. They modified the Walker model and adopted the Forman equation as the basic formula for their model; the equation is expressed as follows:

(8) $$\frac{da}{dN}=\frac{A\Delta K^{m}}{\left[\left(1-R\right)K_{IC}-\Delta K\right]}$$

where *A* is a constant and *K_IC_* denotes the general fracture toughness. While the proposed model effectively predicts the fatigue crack growth behavior, it does not account for the influence of loading frequency.

**Figure 13:**
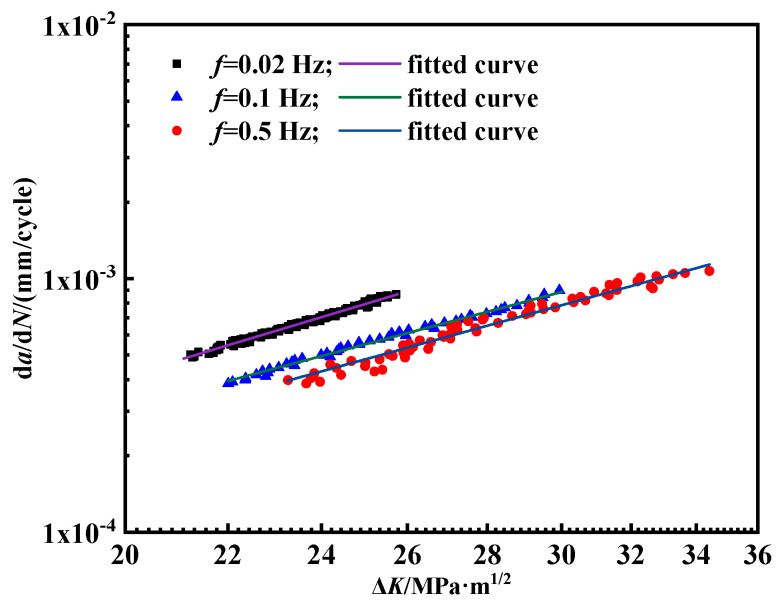
Fitting of d*a*/d*N*-Δ*K* curves of Cheng’s model.

Based on the above equation, the fatigue crack growth trend of pipeline steel under a hydrogen pressure of 2 MPa is estimated. Figure 13 illustrates the corresponding fitted curves for the various frequencies; Table 4 presents the values of *B* and *m* in the formula under different conditions. All the fitted values in Figure 12 are predicted by Equation (8). The fitting curve for a frequency of 0.5, calculated using Origin, has an r value of 0.9772, while the fitting curves for frequencies of 0.1 and 0.02 have r values of 0.9910 and 0.9895, respectively.

As shown in Figure 13, even though the fitting curve matches the experimental data well, varied B and m values are applied in the prediction. Table 4 presents the *B* and *m* values and notable variations in these parameters are observed across different operating conditions. Consequently, the equation is not suitable for accurately predicting the crack growth rate in pipelines under varying frequencies.

Walker’s formula and the modified formula involve the effect of the stress ratio but do not consider the effect of the frequency. The frequency effect was introduced into the combined factor model [45]. In this model, the change in stress intensity is quantified by Δ*K* and *K*_max_, instead of Δ*K* and *R.*

The equation is expressed as follows:

(9) $$\frac{da}{dN}=A{\left(\frac{\Delta K^{2}K_{\max}}{f^{0.1}}\right)}^{b}$$

**Figure 14:**
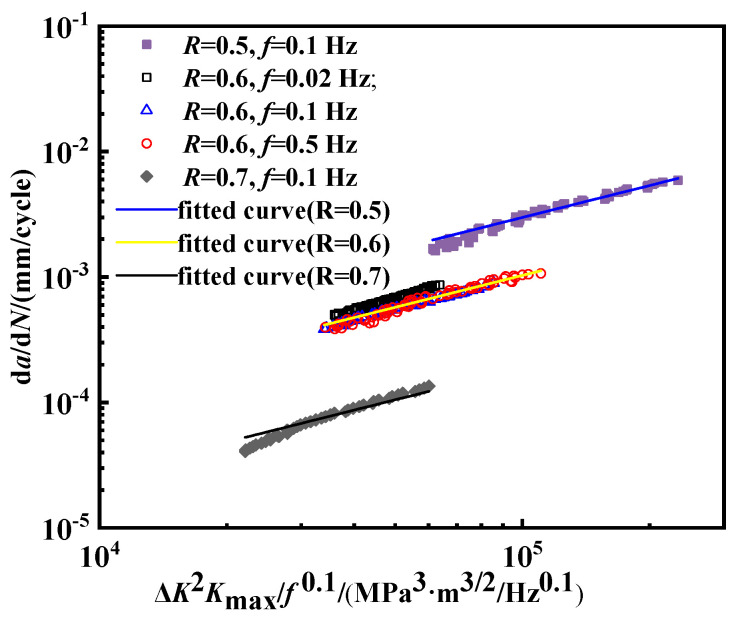
Fatigue crack growth curve described by Δ*K*^2^*K*_max_/*f*^0.1^.

According to the above equation, the fatigue crack growth curves under 2 MPa hydrogen pressure are depicted as shown in Figure 14. It is observed that the predictive curves exhibit good overlap for different frequencies when *R* is 0.6. However, when the *R* value is different the coincidence of the curves is poor and the fitted values of *A* and *b* in the formula are quite different, as shown in Table 5. Thus, the combined factor model is not applicable in the case of different *R* values. All the fitted values in Figure 13 are predicted by Equation (9).

Building on the Walker formula and incorporating a combined factor model, a revised fatigue crack propagation model is introduced as follows:

(10) $$\frac{da}{dN}=A^{\prime }\left[\frac{\Delta K^{2}K_{\max}}{f^{0.1}{\left(1-R\right)}^{1-r}}\right]$$

To solve for the constants in the model, the equation is transformed into the logarithmic form which is as follows:

(11) $$\mathrm{lg}\frac{da}{dN}=b^{\prime }\mathrm{lg}\left(\frac{\Delta K^{2}K_{\max}}{f^{0.1}}\right)-b^{\prime }\left(1-r\right)\mathrm{lg}\left(1-R\right)+\mathrm{lg}A^{\prime }$$

According to the experimental data, the variables in Formula (11) are determined using the multiple linear regression method, where *b*′ is 0.88, *r* is 7.02, and *A*′ is 5.44 × 10^−6^. The fatigue crack growth rate model for pipeline steel under a hydrogen pressure of 2 MPa is then expressed as the following:

(12) $$\frac{da}{dN}=5.44\times 10^{-6}{\left[\frac{\Delta K^{2}K_{\max}}{f^{0.1}{\left(1-R\right)}^{-6.02}}\right]}^{0.88}$$

**Figure 15:**
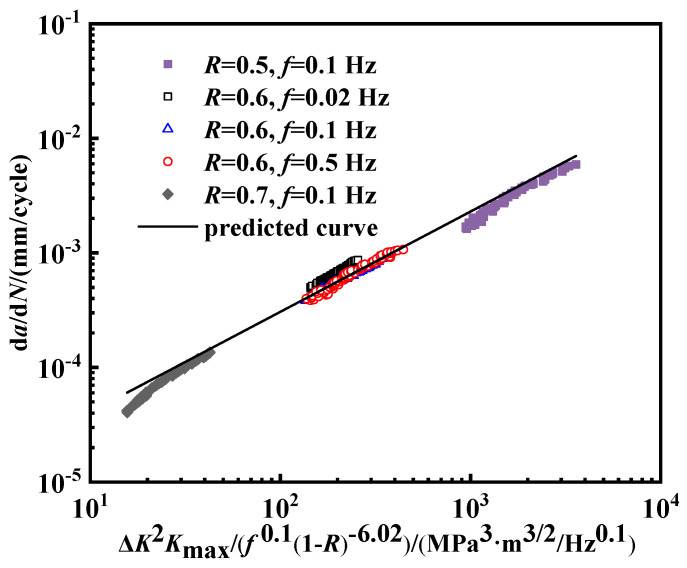
Predictive model under a specific hydrogen pressure.

Taking Δ*K*^2^*K*_max_/(*f*^0.1^(1 − *R*)^−6.02^) as the *x*-axis, the fatigue crack propagation curves are depicted. As presented in Figure 15, the model matches the experimental data well. The fitted values in Figure 14 are predicted by Equation (12).

To incorporate the effect of hydrogen pressure, a linear summation model can be used. Amaro et al. formulated a constitutive model that combines both material deformation and hydrogen diffusion processes, forming the basis for a phenomenological model to predict fatigue crack growth in high-pressure hydrogen environments. The model is expressed as follows:

(13) $${\frac{da}{dN}}_{total}={\frac{da}{dN}}_{fatigue}+\delta \left(P_{H}-P_{H\;th}\right){\frac{da}{dN}}_{H}$$

where the first term in the equation is represented by the Paris relationship, with *δ* being the Dirac delta operator, and *P_Hth_* denoting the threshold hydrogen pressure below which hydrogen-assisted fatigue crack growth (HA FCG) is not observed. This model emphasizes the importance of deformation mechanisms in the fatigue crack growth of pipeline steels under field conditions. However, it does not consider the effects of varying stress ratios and loading frequencies on crack propagation [46].

Based on the Amaro model and our developed model under specific hydrogen pressure, a new model is formulated as follows:

(14) $$\frac{da}{dN}=\frac{da}{dN_{th}}+\frac{da}{dN_{P}}$$

In Equation (14), the term (da/d*N*)_th_ represents the fatigue crack growth rate below the hydrogen pressure threshold, referencing the model established by Amaro et al. [46], and so the hydrogen pressure threshold is set at 0.02 MPa. The term (d*a*/d*N*)_P_ indicates the effect of hydrogen gas pressure on the rate of crack propagation. Consequently, the predictive model is as follows:

(15) $$\frac{da}{dN}=A_{1}Z^{b_{1}}+A_{2}Z^{b_{2}}{\left(P_{H}-P_{th}\right)}^{b_{3}}$$

(16) $$Z=\frac{\Delta K^{2}K_{\max}}{f^{0.1}{\left(1-R\right)}^{-6.02}}$$

where the parameters associated with the load are represented by the factor *Z*, and *P*_H_ and *P*th denote hydrogen partial pressure and hydrogen pressure threshold, respectively. In Equation (15), *A*_1_ is determined as 6.28 × 10^−7^ and *b*_1_ is 1.14. To determine the values of other constants in the model, a nonlinear surface fitting is performed using the least squares method. The obtained values are *A*_2_ = 4.27 × 10^−6^, *b*_2_ = 0.21, and *b*_3_ = 4.61. Thus, the fitted model could be shown as:

(17) $$\frac{da}{dN}=6.28\times 10^{-7}Z^{1.14}+4.27\times 10^{-6}Z^{0.21}{\left(P_{H}-0.02\right)}^{4.61}$$

(18) $$Z=\frac{\Delta K^{2}K_{\max}}{f^{0.1}{\left(1-R\right)}^{-6.02}}$$

**Figure 16:**
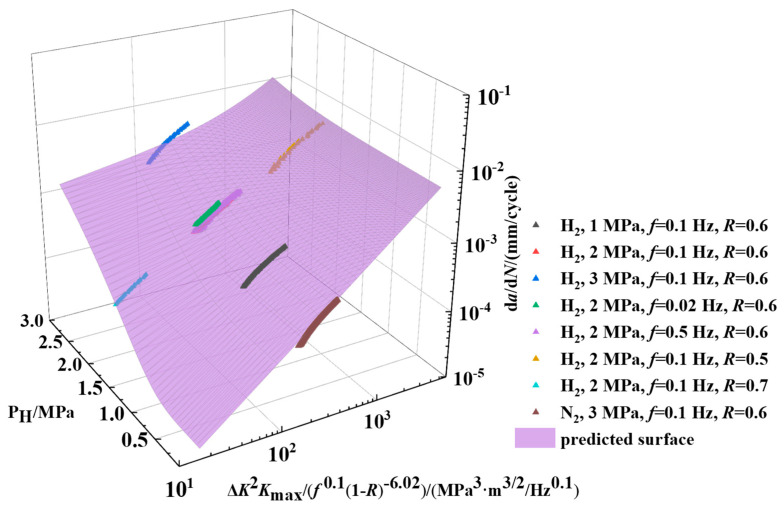
Comparison of predictive values with experimental results under different loading conditions and hydrogen pressures.

Figure 16 shows the results of the model prediction. All the fitted values in Figure 16 are predicted by Equation (17). The fitted model matches well with experimental results under different loading conditions and hydrogen pressures below 3 MPa. Based on the experimental results shown in Figure 16, a comparison is made between the predicted results from Equation (17) and the actual experimental data. Table 6 presents the average relative errors under different operating conditions. Currently, the hydrogen blending ratio in the gas pipeline barely exceeds 20%. Since the natural gas transporting pressure is below 10 MPa, the partial hydrogen pressure remains under 2 MPa [47]. Therefore, the fatigue crack growth rate model remains applicable for estimating the fatigue life of hydrogen-blended X80 pipeline steel. This model is applicable to all conditions with hydrogen pressure below 3 MPa. However, when the pressure increases, the value of *b_3_* in Equation (15) can be modified to adjust the form of Equation (17) for predicting crack growth under those conditions. Similarly, when using different types of steel, the values of *A*_1_, *A*_2_, *b*_1_, and *b*_2_ can be adjusted to derive a new equation for the respective material. However, when hydrogen pressure exceeds 3 MPa, the fatigue crack growth rate increases significantly, approximately an order of magnitude greater than the rate observed without hydrogen. Hence, further investigation is needed to understand the mechanism of crack propagation under higher hydrogen pressures in X80 pipeline steel. This study identifies the influence of different hydrogen pressures and loading conditions on the fatigue crack growth behavior of hydrogen transport pipeline steel. Based on the experimental results, a crack propagation prediction model has been developed, which can be applied for the life prediction of hydrogen transportation pipeline steel.

## 5. Conclusions

This study investigated the fatigue crack propagation under moderate hydrogen gas pressure conditions. The crack surface features are observed, and a model is established to predict crack growth rates in X80 steel under different hydrogen pressure and loading interactions. The following conclusions are drawn:

(1) The presence of hydrogen significantly increased the fatigue crack propagation rate of X80 pipeline steel. When the hydrogen pressure is 3 MPa, the fatigue crack propagation rate in X80 steel increased by one order of magnitude compared to that in air, reaching 10^−2^ mm/cycle. The stress intensity factor threshold for crack growth in nitrogen is 20 MPa·m^1/2^, but hydrogen lowers the stress intensity threshold, Δ*K*_th_, below which the crack cannot propagate. The presence of hydrogen reduces the interatomic bonding, particularly at grain boundaries or other defects at the crack tip. This facilitates cleavage fracture, significantly lowering the crack growth threshold and accelerating crack propagation.

(2) In a hydrogen environment, the fatigue crack growth rate per cycle is highly sensitive to the loading frequency. With an increase in the loading frequency, the crack propagation rate decreases. When the frequency is 0.02 Hz, the crack propagation rate reaches a maximum of 10^−2^ mm/cycle, whereas at 0.5 Hz, the fatigue crack propagation rate is mostly below 10^−3^ mm/cycle. A lower frequency results in longer loading cycles, providing more time for hydrogen atoms to diffuse and concentrate at the crack tip, creating a hydrogen-enriched zone that accelerates fatigue crack growth.

(3) The fatigue crack growth rate increases with the decrease in the stress ratio. When the stress ratio reaches 0.5, the crack propagation rate can rise to a maximum of 10^−1^ mm/cycle. Under low stress ratios, the stress range is large and the crack growth is enhanced by the accumulation of at the crack tip. Also, hydrogen atoms tend to diffuse to the crack tip and further enhance the crack growth at a low stress ratio.

(4) The presence of hydrogen significantly alters the morphology of fatigue fracture, inducing brittle characteristics. As no hydrogen exists, the fracture surface is ductile and covered with dimples. A river pattern fracture morphology and tear edges are observed in the hydrogen environment. In the final stage of hydrogen-induced cracking, secondary cracks and sizeable brittle facets are observed at the fracture surface.

(5) Based on experimental data, the Walker equation, a combined factor model, and Amaro’s constitutive model are correlated to generate a predictive model for fatigue crack propagation in pipeline steel under different hydrogen pressure and loading interactions. The predictive values agree very well with the experimental results. The average relative errors between predictive values and experimental data are limited below 10%.

## Figures and Tables

**Table 1:** Chemical composition of X80 pipeline steel (mass fraction, %).

C	Si	Mn	Cu	Mo	N	P	Ni	Ti	Cr	Al	Nb	S
0.06	0.12	1.42	0.02	0.18	0.003	0.017	0.03	0.096	0.05	0.026	0.03	0.002

**Table 2:** Value of parameters in Equation (2).

*C*_0_	*C*_1_	*C*_2_	*C*_3_	*C*_4_	*C*_5_
1.0002	−4.0632	11.242	−106.04	464.33	−650.68

**Table 3:** Characteristic constants of the Paris formula under different conditions.

Frequency	Stress Ratio	Hydrogen Pressure	*C*	*m*
0.1 Hz	0.6	1 MPa	4.36 × 10^−8^	2.81
0.1 Hz	0.6	2 MPa	1.25 × 10^−7^	2.61
0.1 Hz	0.6	3 MPa	1.19 × 10^−7^	3.03
0.02 Hz	0.6	2 MPa	6.29 × 10^−8^	2.93
0.1 Hz	0.6	2 MPa	1.25 × 10^−7^	2.61
0.5 Hz	0.6	2 MPa	8.28 × 10^−8^	2.69
0.1 Hz	0.5	2 MPa	1.82 × 10^−7^	2.74
0.1 Hz	0.6	2 MPa	1.25 × 10^−7^	2.61
0.1 Hz	0.7	2 MPa	3.79 × 10^−9^	3.29

**Table 4:** Characteristic constants of Formula (8).

Frequency	Stress Ratio	Hydrogen Pressure	*A*	*m*
0.02 Hz	0.6	2 MPa	5.64 × 10^−7^	3.52
0.1 Hz	0.6	2 MPa	1.30 × 10^−2^	−1.20 × 10^−2^
0.5 Hz	0.6	2 MPa	3.72 × 10^−2^	−3.03 × 10^−2^

**Table 5:** Characteristic constants of Formula (9).

Stress Ratio	Frequency	Hydrogen Pressure	*A*	*b*
0.5	0.1 Hz	2 MPa	7.85 × 10^−8^	0.91
0.6	0.02 Hz	2 MPa	1.73 × 10^−8^	0.98
0.6	0.1 Hz	2 MPa	4.25 × 10^−8^	0.87
0.6	0.5 Hz	2 MPa	3.38 × 10^−8^	0.90
0.7	0.1 Hz	2 MPa	7.85 × 10^−10^	0.10

**Table 6:** Average relative errors between predicted and experimental values under different operating conditions.

Stress Ratio	Frequency	Hydrogen Pressure	Error
0.6	0.1 Hz	1 MPa	5.7%
0.6	0.1 Hz	2 MPa	3.3%
0.6	0.1 Hz	3 MPa	4.8%
0.6	0.02 Hz	2 MPa	2.5%
0.6	0.5 Hz	2 MPa	3.7%
0.5	0.1 Hz	2 MPa	5.1%
0.7	0.1 Hz	2 MPa	4.8%
0.6	0.1 Hz	3 MPa (N_2_)	6.3%

## Data Availability

The data that have been used are confidential.

## Nomenclature

BCC	body center cubic
HE	hydrogen embrittlement
*f*	frequency (Hz)
*R*	stress ratio
Δ*K*	Range of change in stress intensity factor(MPa·m^1/2^)
SEM	scanning electron microscope
EDS	energy dispersive spectroscopy
CT	compact tensile
PF	quasi-polygonal ferrite
AF	acicular ferrite
GB	granular bainite
*U_x_*	the dimensionless compliance
*B*	the specimen thickness (mm)
*E*	the modulus of elasticity (MPa)
*V_x_*	the displacement observed at the designated measurement point (mm)
*F*	the force loaded on the specimen (N)
*a*	the crack length (mm)
*W*	the width of the specimen (mm)
*C*_0_~*C*_5_	constant
*N*	number of cycles of fatigue loading
*da/dN*	crack growth rate (mm/cycle)
*g* (*a/W*)	the specimen geometry factor
*Fmax*	the maximum applied force (N)
Δ*K*_th_	fatigue crack growth threshold (MPa·m^1/2^)
*m*	material-dependent constant
*n*	constant
*A*	constant
*K_IC_*	general fracture toughness (MPa·m^1/2^)
*K_max_*	maximum stress intensity factor (MPa·m^1/2^)
*r*	constant
*b*′	constant
*δ*	the Dirac delta operator
*P_H_*	partial pressure of hydrogen (MPa)
*P*th	hydrogen pressure threshold (MPa)
HA	hydrogen-assisted
FCG	fatigue crack growth
